# Individual psychotherapy may help to reduce suicidal ideation in first episode psychosis: results from a 2-year Italian follow-up study

**DOI:** 10.1192/j.eurpsy.2023.1185

**Published:** 2023-07-19

**Authors:** L. Pelizza, E. Leuci, E. Quattrone

**Affiliations:** Department of Mental Health, AUSL di Parma, Parma, Italy

## Abstract

**Introduction:**

Suicidal thinking is relevant in patients with First Episode Psychosis (FEP). However, longitudinal studies specifically examining treatment response for suicidal ideation in FEP are still relatively scarce, especially with long-term design and in real-world clinical settings.

**Objectives:**

The aims of this research were (A) to longitudinally assess suicidal thoughts in people with FEP along a 2-year follow-up period and (B) to overtime investigate any significant association of suicidal ideation levels with the specific treatment components of an ‘Early Intervention in Psychosis’ (EIP) protocol along the 2 years of follow-up.

**Methods:**

At entry, 232 FEP participants (aged 12–35 years) completed the Brief Psychiatric Rating Scale (BPRS), including a ‘Suicidality’ item subscore. Multiple linear regression analysis was then performed.

**Results:**

Across the follow-up, FEP subjects showed a relevant decrease in suicidal thinking levels overtime. This was specifically predicted by the total number of individual psychotherapy sessions offered within the 2-year EIP protocol and antidepressant dose (at least as regards the first year of our intervention).

**Image:**

**Figure:**
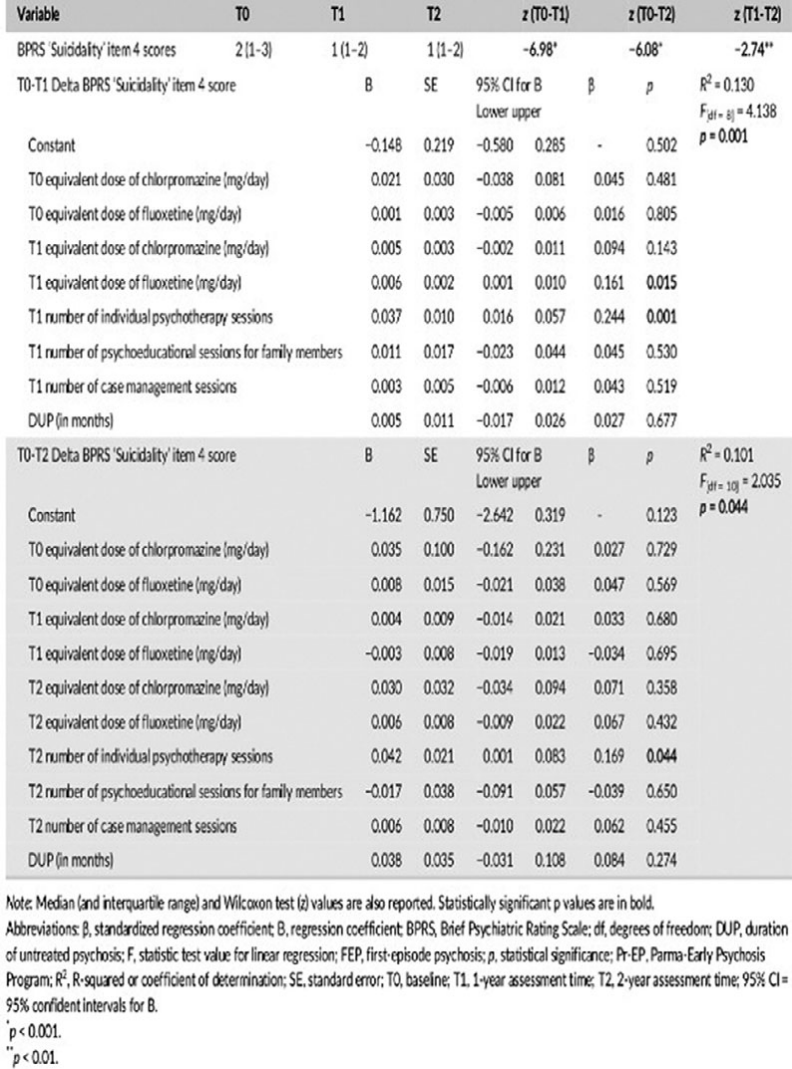

**Conclusions:**

Suicidal ideation is clinically relevant in FEP but seems to improve overtime together with the provision of specific, patient-tailored and integrated EIP treatments, especially individual psychotherapy.

**Disclosure of Interest:**

None Declared

